# Supplementary material for: Epigenetic maintenance of PRC2-repressed chromatin requires RTT109 but not H3K56 acetylation
Source: PLoS Genet. 2026 Oct 5;22(10):e1012327. doi: 10.1371/journal.pgen.1012327 (PMC13652317; doi:10.1371/journal.pgen.1012327)
Supplement: Supplementary file 1 [file pgen.1012327.s009.docx]

| S1 Table. List of primers used in this study. | | |
| --- | --- | --- |
| Primer name | Sequence | Purpose |
| twist fp | CAATCCGCCCTCACTACAACCG | Used to amplify catalytic-dead rtt109 constructs to put into pBM61 |
| twist rp | TCCCTCATCGACGCCAGAGTAG | Used to amplify catalytic-dead rtt109 constructs to put into pBM61 |
| RY59-Gibson-Inverse-Primer-2 | GTGGACGGCTAATGGGGTCTGAATGCTAAA | Used to amplify pBM61 backbone for csr-1 integrations |
| RY99-pBM61GibsonInverseExtended | AGCTCCAATTCGCCCTATAGTGAGTCGTATTAAGG | Used to amplify pBM61 backbone for csr-1 integrations |
| NCU04869_qPCR_F | TGGTGCGTTCAACCTGAAAA | Used for ChIP-qPCR |
| NCU04869_qPCR_R | TCACCATTTTCTTCCGCACAA | Used for ChIP-qPCR |
| RYq7-ncu06889-F | CATCTTGTATCGAACCCCTTGC | Used for ChIP-qPCR |
| RYq8-ncu06889-R | AAGAGATCGAGGAATGTACGGC | Used for ChIP-qPCR |
| gh3-6-R-qPCR | CAAGAGGCAGGGTGGTGTAAT | Used for ChIP-qPCR |
| gh3-6-F-qPCR | TGCCCATCAATCCCAAGACC | Used for ChIP-qPCR |
| 5 prime landing pad FP | CAACAAGCACATCCAGACACTGAG | Used to create ncu06889::bar |
| 3 prime landing pad RP | CTCTTCTCATGGCCATGTTGATTC | Used to create ncu06889::bar |
| RY49-rtt109-R | CACTTTGTACACACTACACA | Used to genotype rtt109 |
| RY50-rtt109-F | TGTATGGTGAAGCAAAAGCT | Used to genotype rtt109 |
| SUZ12rev | GGGTTGTGCGAGGTGTGG | Used to make tetR-suz12-gfp |
| SUZ12fwd | TCCGTCTTTTGGGTAGCTAGAT | Used to make tetR-suz12-gfp |
| csr1 3p RP w overhang NEW | AATTAATACGACTCACTATAGGGAGGCCCCTGGTTTACTGAGGGC | Used to integrate tetR-suz12-gfp into csr-1 |
| csr1 5 FP overhang | CACTATAGAACTCGAGCAGCTGAAGCAACACCTCCGTCGCCATAAACTCC | Used to integrate tetR-suz12-gfp into csr-1 |
| RY137 Gibson-csr-1-rtt109-3'flank | GATAAGCTTGATATCGAATTCTTAGCATCCTCGTCGACGACCCCTGCCCC | Used to genotype rtt109 integrations in csr-1 locus |
| RY138 Gibson-csr-1rtt109-5'flank | CACTAGTTCTAGAGCGGCCGCTAAGCTGACGTTTTGGGCTTCTTCCTGAT | Used to genotype rtt109 integrations in csr-1 locus |
| RTT109 3'UTR | TGTGGGAGAAGGTGATGTTACAAATC | Used to create knock-in rtt109-3xflag |
| RTT109-3'CDS-3xflag | CCTCCGCCTCCGCCTCCGCCGCCTCCGCCAGCTGACGTTTTGGGCTTCTTCC | Used to create knock-in rtt109-3xflag |
| RTT109-5' CDS-F | AGTCAAGAAACACCAACGGAATTACC | Used to create knock-in rtt109-3xflag |
| RTT109-5'UTR-3xflag | TATTCTATAGTGTCACCTAAATAGCTTGGGTCCTCATGATTACTCTTTTCTTTCTTAGTC | Used to create knock-in rtt109-3xflag |
