# Supplementary material for: Epigenetic maintenance of PRC2-repressed chromatin requires RTT109 but not H3K56 acetylation
Source: PLoS Genet. 2026 Oct 5;22(10):e1012327. doi: 10.1371/journal.pgen.1012327 (PMC13652317; doi:10.1371/journal.pgen.1012327)
Supplement: Supplementary file 2 [file pgen.1012327.s010.docx]

| S2 Table. List of strains used in this study | |  |
| --- | --- | --- |
| Strain # | Genotype | Source |
| S2 | Wild type *N. crassa* (FGSC2489) | FGSC, Kansas City |
| S542 | his-3+::tetO; mus-52::bar+ | 1 |
| S543 | his-3+::tetO; csr-1::p[TrpC]::TetR::eGFP ; mus-52::bar+ | 1 |
| S872 | his-3::tetO, csr-1::p[Dim5]-tetR-suz12-gfp; mus-52::bar+ | This study |
| S866 | ncu06889::bar | This study |
| S858 | rtt109::rtt109-3xflag-hph | This study |
| S8 | mus-52::bar+ | 2 |
| S11 | mus-52::hph+ | 2 |
| S337 | rtt109::hph, mus-51::hph | This study |
| S534 | rtt109::hph | This study |
| S857 | rtt109::hph (Parental) | This study |
| S603 | eed::hph | This study |
| FGSC12340 | rtt109::hph | FGSC, Kansas City |
| FGSC11778 | naf-2::hph | FGSC, Kansas City |
| S539 | asf-1::hph-KD | This study |
| S955 | H3K56R | 3 |
| S957 | H3K56Q | 3 |
| S238 | set-7::hph | This study |
| S960 | eed::hph, ncu06889::bar | This study |
| S961 | rtt109::hph, ncu06889::bar | This study |
| S962 | rtt109::hph Passage 0 | This study |
| S963 | rtt109::hph Passage 1 | This study |
| S964 | rtt109::hph Passage 2 | This study |
| S965 | H3K56R Passage 0 | This study |
| S966 | H3K56R Passage 3 | This study |
| S967 | rtt109::hph, mus-51::hph, csr-1::rtt109-3xflag | This study |
| S968 | rtt109::hph, mus-51::hph, csr-1::rtt109-D145A-3xflag | This study |
| S969 | rtt109::hph, mus-51::hph, csr-1::rtt109-D145A-DD304-305AA-3xflag | This study |
| S970 | rtt109::hph, his-3::tetO, csr-1::p[Dim5]-tetR-suz12-gfp | This study |
| S1019 & 1020 | ∆naf-2::hph, rtt109::rtt109-3xflag | This study |
