# Supplementary material for: Epigenetic maintenance of PRC2-repressed chromatin requires RTT109 but not H3K56 acetylation
Source: PLoS Genet. 2026 Oct 5;22(10):e1012327. doi: 10.1371/journal.pgen.1012327 (PMC13652317; doi:10.1371/journal.pgen.1012327)
Supplement: Supplementary file 3 [file pgen.1012327.s011.docx]

| Supplemental Table 3. Antibodies used in this study | | | |
| --- | --- | --- | --- |
| Antibody | Host | Supplier | Catalog number |
| H3K27me3 | Mouse | Diagenode (Novus) | NBP2-59295 |
| Anti-FLAG M2 | Mouse | Sigma | F1804-200UG |
| H3K27me3 | Mouse | Abcam | ab6002 |
| H3K36me3 | Rabbit | Abcam | ab9050 |
| Histone H3 | Rabbit | Active Motif | 61799 |
| Anti-mouse IgG (H+L) HRP Conjugate | Goat | ThermoFisher | 31450 |
| Anti-Rabbit IgG HRP Conjugate | Goat | Active Motif | 15015 |
| H3K27me3 | Rabbit | Cell Signaling Technology | C36B11 |
| H3K9me3 | Rabbit | Active Motif | 39161 |
