# Supplementary material for: Epigenetic maintenance of PRC2-repressed chromatin requires RTT109 but not H3K56 acetylation
Source: PLoS Genet. 2026 Oct 5;22(10):e1012327. doi: 10.1371/journal.pgen.1012327 (PMC13652317; doi:10.1371/journal.pgen.1012327)
Supplement: Supplementary file 4 [file pgen.1012327.s028.docx]

**Editor Remarks to Author:**
Thank you for submitting this very interesting and well done manuscript to PLOS Genetics. The manuscript was reviewed by three reviewers, all experts in the field. You will be glad to hear that they found the work interesting and well done, and suggest minor revisions and some edits to consider alternatives. Therefore, we invite you to submit a revised version of the manuscript that addresses the points raised during the review process. The revisions needed constitute minor revision.

Please submit your revised manuscript within two months (it may not take this long). But if you will need more time than this to complete your revisions, please reply to this message or contact the journal office at plosgenetics@plos.org. When you're ready to submit your revision, log on to https://www.editorialmanager.com/pgenetics/ and select the 'Submissions Needing Revision' folder to locate your manuscript file.

We thank the reviewers for their constructive comments and suggestions. We have performed additional experiments and revised the manuscript in response. We have also made editorial changes requested by the journal office. We believe the manuscript is much improved as a result. Our response to each reviewer comment is detailed below.

Reviewer Comments:

**Reviewer #1:**
Comments:
This manuscript identifies RTT109 as a regulator of facultative heterochromatin maintenance in Neurospora crassa and demonstrates that this function is independent of NAF-2 and H3K56 acetylation. The authors combine genetics, transcriptomics, chromatin profiling, protein interaction analyses, and an elegant synthetic recruitment system to investigate how H3K27me3 domains are maintained through mitotic divisions. The work addresses an important question in chromatin biology and provides convincing evidence that RTT109 contributes to maintenance, rather than establishment, of facultative heterochromatin.

The experiments are well designed and the conclusions are generally supported by the data. In particular, the synthetic tetO-SUZ12 recruitment system provides strong evidence that H3K27me3 can be maintained in a PRC1-independent fungal system and that RTT109 is required for this process.

**Major comments**

**The authors show that RTT109 regulates facultative heterochromatin independently of NAF-2 and H3K56 acetylation. This result raises an obvious mechanistic question: which molecular form of RTT109 carries out this function? The data exclude the canonical RTT109–NAF-2–H3K56ac pathway, but they do not address whether RTT109 acts as a free protein, in association with NAF-2, or as part of another protein complex. Because RTT109 co-purifies with NAF-2 whereas NAF-2 itself is dispensable for maintenance of facultative heterochromatin, the possibility that RTT109 participates in additional protein complexes deserves consideration. The authors could address this point experimentally using the existing RTT109-3xFLAG strain. Native size-exclusion chromatography or glycerol gradient fractionation followed by anti-FLAG immunoblotting would determine whether RTT109 exists in multiple biochemical pools. Analysis of NAF-2 co-fractionation would help distinguish the canonical RTT109–NAF-2 complex from potential NAF-2-independent assemblies. In addition, RTT109 immunoprecipitation in a naf-2Δ background could establish whether (i) Rtt109 interacts to heterochromatic domains and (ii) this interaction is dependent on its canonical protein complexes. I do not consider identification of the relevant RTT109 substrate necessary for publication. However, a simple biochemical characterization of RTT109 complexes would strengthen the mechanistic interpretation of the study.**

We constructed an ∆*naf-2 rtt109-3xflag* strain and carried out IP LC-MS/MS. This did not identify additional interacting proteins. We now include these results as supplemental data (Supplementary Fig. 4D, Supplementary Data Tabs 11,12). We have also added the following lines to the text:

Purification of RTT109 from a ∆naf-2 mutant failed to identify additional RTT109-interacting proteins that were enriched over background levels and might modulate substrate specificity (Supplementary Fig. 4D, Supplementary Data Tab 11,12).

**2. The effects of RTT109 loss on facultative heterochromatin-associated H3K36me3 are interesting and may provide insight into the underlying mechanism. The passaging experiments suggest that loss of H3K36me3 occurs before the progressive loss of H3K27me3. The authors should discuss this possibility in greater detail.**

We agree that H3K36me3 loss could be an underlying factor leading to H3K27me3 loss. We added the following lines to the discussion:

“H3K36me3 is rapidly lost in early passages of the *∆rtt109* mutant, raising the possibility that RTT109 regulates activity or localization of the ASH1-dependent H3K36 methyltransferase. Indeed, given that loss of H3K36me3 within facultative heterochromatin precedes the progressive loss of H3K27me3, it is possible that ASH1-dependent H3K36me3 is critical for maintaining H3K27me3 over sequential cell division cycles [39, 41].”

**If feasible, analysis of ASH1 occupancy (by ChIP-seq/ChIP PCR) or ASH1 protein levels in the Δrtt109 strain would help clarify this point.**

This is a great suggestion. Unfortunately, we have not been able to localize ASH1 by performing ChIP-seq using an ASH1-3xFLAG strain. Prior studies have indicated that extended residence times are required for efficient protein/DNA crosslinks (Schmiedeberg et al. 2009. *PLoS One*). It is therefore likely that ASH1 interacts transiently with chromatin.

**3. In the introduction, the authors state that RTT109 plays a role in quelling and small-RNA production in N. crassa. However, the discussion does not revisit this aspect of RTT109 biology. The authors should discuss whether the previously reported roles of RTT109 in d small-RNA pathways could be related to the chromatin phenotypes described here, or whether they consider these functions to be independent.**

This is a good point. We have added the following to the discussion*:*

Both RTT109 and H3K56 acetylation are further required for proper repair of DNA double strand breaks by homologous recombination and for production of small interfering RNAs in *N. crassa* [45, 53, 71]*.* Our findings reveal that RTT109 plays an additional, mechanistically distinct role in maintaining facultative heterochromatin in filamentous fungi*.*

**Reviewer #2**

Comments:
 The authors present a comprehensive analysis of RTT109 in N. crassa and show that it is required for the establishment of facultative heterochromatin (fHC) at several regions in the genome. The manuscript reports several important novel findings related to the role of RTT109 in the establishment and maintenance of fHC over mitotic divisions. Latter has been tested by the implementation of an innovative system using a synthetic TetR-suz12 fusion which allows to target the PRC2 complex conditionally to a TetO array inserted at the his3 locus. They also demonstrated that the acetyltransferase activity of RTT109 is necessary for its PRC2 recruitment function but, surprisingly, the target is not histone H3K56. Apparently, something else must be acetylated by RTT109 to mediate recruitment of PRC2 components. The identity of this RTT109 target remains elusive. In any case, all necessary experimental controls were considered and the manuscript is well structured. In my view, great work from one of the leading labs in fungal chromatin biology and epigenetics. **There are just a few points for the authors to consider:
1. Fig 1 shows that RTT109 is required for the establishment of H3K27me3 domains and concomitant transcriptional repression but quantification by MACS3 only reveals a mild effect (281 region in WT versus 206 regions in RTT109 KO corresponding to a decrease of 29%). As the affected regions are known, it might be interesting to search for position effect biases that define RTT109-targeted regions (e.g. subtelomeric or pericentric regions bias?). This could be checked and if any significant enrichments appear, amended the manuscript with these data.**

Thank you for the suggestion. We performed additional analyses to examine possible position effect biases in ∆*rtt109,* which revealed a strong bias for regions distal to the telomere. These data are now provided in Supplemental Figure 2E-G.

We added the following text to describe these results:

“Telomeres are known to direct *de novo* establishment of H3K27me3 [31, 49]. The H3K27me3-enriched peaks lost in the ∆*rtt109* strain were predominantly located more than 200kb from the telomere (78.9%; Supplementary Fig. 2E). Consistent with this pattern, domains located near telomeres retained H3K27me3 or exhibited enrichment relative to wild type, whereas the magnitude of H3K27me3 loss increased with distance from the telomere (Supplementary Fig. 2F). Nevertheless, distance from the telomere was not an absolute predictor of H3K27me3 loss, as some distal regions retained near-wild-type levels of H3K27me3 (Supplementary Fig. 2F,H).”

**2. Related to this possible positional bias are the results presented in Fig 5 in which the role of RTT109 in H3K27me3 maintenance during mitotic divisions was tested. Actually, H3K27me3 deposition at the TetO array inserted at the his 3 locus is not RTT109 dependent. Only the maintenance and mitotic stability is. One interpretation consistent with the data is that RTT109 is necessary only for PRC2 recruitment, but not for its function. If recruitment occurs via TetOFF-suz12, then RTT109 is not necessary anymore (K27me3 equally strong in WT and mutant background, Fig 5 E/F). I think this should be more clearly stated in the discussion.**

This is a good point. We have added the following text to the discussion:

“Our data suggest that RTT109 is not required for PRC2 catalytic activity *per se* or for de novo methylation at strong recruitment sites. In the ∆rtt109 strain, we observed elevated H3K27me3 levels near telomeres, which are known to be strong PRC2 recruitment sites [31, 49]. In addition, tethering the tetR-GFP-SUZ12 construct to tetO produced high levels of H3K27me3 in both the wild type and ∆*rtt109* strains.”

**Also, possible candidates that are known to affect H3K27 methylation, could be checked for the acetylation status in the published dataset of Wang et al (2021). PRC2 components were not found, but were other factors known to affect K27 methylation checked?**

Yes, additional factors were checked. The relevant text in the discussion is: “Several known or potential regulators of facultative heterochromatin were found to be acetylated including the chromatin remodeler ISW, the histone deacetylase RPD3, a putative ASH1-interacting protein, and others. Testing these acetylated residues for possible roles in facultative heterochromatin is an important goal for future studies.”

**3. Fig 3 G: the loading of the lanes for the Western blot does not look balanced as the H3 band is much weaker in the rtt109 mutant. I suggest a densitometric analysis of serial dilutions that should allow quantification of H3K27 methylation in the different genetic backgrounds.**

We repeated three additional biological replicates of the H3K27me3 western blot experiment using a dilution series of 10, 5, and 2.5 µg of protein for WT, ∆set-7, ∆rtt109, hH3^K56R^, hH3^K56Q^. The quantified signal for each strain is shown above the Western blot in figure 3G.

**Minor points:
The authors should mention in the introduction that H3K27 methylation also regulates secondary metabolite production in fungi and that many filamentous ascomycetes do not contain the PRC2 machinery to establish fHC this way.**

We have added the following to the introduction:

In some fungi, PRC2 components are present and have been linked to multicellular development, secondary metabolite production, stress responses, and virulence [7-11].

We also revised the following statement: “Many filamentous fungi assemble PRC2-dependent facultative heterochromatin associated with stable repression of condition-specific genes, but PRC1 was apparently lost in an early fungal ancestor and PRC2 is absent from some fungal lineages [26]*.*”

**Reviewer #3**

Comments:
The study identifies a novel role for the histone acetyltransferase RTT109 in regulating PRC2-mediated facultative heterochromatin. RTT109 is primarily known for its roles in replication-coupled nucleosome assembly and DNA repair through H3K56 acetylation. Its involvement in maintaining H3K27me3-marked facultative heterochromatin was previously unknown, thus expanding its functional repertoire beyond its established roles in euchromatin and DNA damage responses.

Importantly, RTT109 is the main enzyme responsible for H3K56 acetylation in fungi, and most of its known biological functions have been attributed to this modification. The finding that RTT109 maintains H3K27me3 domains through its catalytic activity, yet independently of H3K56 acetylation, therefore suggests a distinct non-canonical mechanism of action. This uncouples its role in facultative heterochromatin from its established H3K56 acetylation-dependent functions.

Furthrermore, although epigenetic inheritance of H3K27me3 across cell divisions is well documented in metazoans, fungi lack the PRC1 complex, which is often critical for this process in higher eukaryotes. The observation that Neurospora crassa can stably propagate H3K27me3 over multiple mitotic divisions therefore provides evidence for a PRC1-independent mechanism for epigenetic memory.

**Few additional considerations:**

**- Maybe this reviewer missed that, but It has not been shown that the tetR-GFP-SUZ12 is fully displaced from the tetO array after tetracycline addition under the conditions used. While tetracycline reliably induces native TetR dissociation through conformational changes, fusion proteins can exhibit altered functional properties compared to their native components, so anti-GFP ChIP-seq followup after tetracycline treatment would be handy.**

This is a good point. We now include data to show that tetracycline leads to dissociation of the tetR-GFP-SUZ12 fusion from the tetO array (Supplementary Fig. 8B).

We included the following text to the results section. “As expected, the tetR-GFP-SUZ12 fusion was no longer detected at the tetO array following addition of tetracycline to the medium (Supplementary Fig. 8B).”

**- This study reports that RTT109 deficiency does not affect constitutive heterochromatin in N. crassa. This result has already been reported in ref. 56.**

We have added the appropriate reference, now: “Our results showed that loss of RTT109 does not alter H3K9me3 patterns relative to wild type, as previously reported, indicating constitutive heterochromatin is regulated independently of RTT109 (Supplementary Fig. 3) [53].”

**- It is still formally possible that RTT109-mediated H3K56Ac may be required for H3K27me3 if one considers a model in which two states exist, “H3K56Ac” and “H3K56”, and “H3K56Ac” represents a “not H3K56” state; in this case replacing H3K56 with anything else will effectively generate a state analogous to “H3K56Ac”.**

This is an interesting idea, and we agree it remains a formal possibility. However, we have opted not to discuss this model here because we feel it’s an unlikely scenario. Given the structural similarity between H3K56R and unacetylated H3K56, we would expect the *hH3K56R* mutant to have some defect in H3K27me3 distribution. In addition, the observation that *rtt109* alleles encoding catalytic null proteins exhibit enhanced phenotypes compared to the ∆rtt109 allele is inconsistent with this model. One interesting prediction of this ‘two-state’ model is that an *hH3K56R* allele would suppress the ∆rtt109 phenotype. This is a good experiment, but one we feel is beyond the scope of the present study. We appreciate this comment and plan to address this with future work.

**Have all data underlying the figures and results presented in the manuscript been provided?**
Large-scale datasets should be made available via a public repository as described in the *PLOS Genetics* [data availability policy](http://track.editorialmanager.com/CL0/http:%2F%2Fjournals.plos.org%2Fplosgenetics%2Fs%2Fdata-availability/1/010f019f14347425-8088ae80-bf20-4fa3-b8a6-afa0ab767e91-000000/MX-EtA2oxs_7UTffte9r5URl7mOzuP_D3ekcQwMD8dA=258), and numerical data that underlies graphs or summary statistics should be provided in spreadsheet form as supporting information.

Reviewer #1: Yes

Reviewer #2: Yes

Reviewer #3: Yes

PLOS authors have the option to publish the peer review history of their article ([what does this mean?](https://track.editorialmanager.com/CL0/https:%2F%2Fjournals.plos.org%2Fplosgenetics%2Fs%2Feditorial-and-peer-review-process%23loc-peer-review-history/1/010f019f14347425-8088ae80-bf20-4fa3-b8a6-afa0ab767e91-000000/W0IJ71gCumcszGU3L2Z7GgYzXpcAop2G0N7UQn2ivvY=258)). If published, this will include your full peer review and any attached files.


If you choose “no”, your identity will remain anonymous but your review may still be made public.


**Do you want your identity to be public for this peer review?** For information about this choice, including consent withdrawal, please see our [Privacy Policy](https://track.editorialmanager.com/CL0/https:%2F%2Fwww.plos.org%2Fprivacy-policy/1/010f019f14347425-8088ae80-bf20-4fa3-b8a6-afa0ab767e91-000000/W0mIPfu7INpTyIv6rthpiR6i8K3yHVnwgqV0x7hShj8=258).

Reviewer #1: No

Reviewer #2: No

Reviewer #3: No

[NOTE: If reviewer comments were submitted as an attachment file, they will be attached to this email and accessible via the submission site. Please log into your account, locate the manuscript record, and check for the action link "View Attachments". If this link does not appear, there are no attachment files.]

**Figure resubmission:**

While revising your submission, we strongly recommend that you use PLOS’s NAAS tool (<https://ngplosjournals.pagemajik.ai/artanalysis>) to test your figure files. NAAS can convert your figure files to the TIFF file type and meet basic requirements (such as print size, resolution), or provide you with a report on issues that do not meet our requirements and that NAAS cannot fix.

After uploading your figures to PLOS’s NAAS tool - <https://ngplosjournals.pagemajik.ai/artanalysis>, NAAS will process the files provided and display the results in the "Uploaded Files" section of the page as the processing is complete. If the uploaded figures meet our requirements (or NAAS is able to fix the files to meet our requirements), the figure will be marked as "fixed" above. If NAAS is unable to fix the files, a red "failed" label will appear above. When NAAS has confirmed that the figure files meet our requirements, please download the file via the download option, and include these NAAS processed figure files when submitting your revised manuscript.

**Reproducibility:**

To enhance the reproducibility of your results, we recommend that authors deposit laboratory protocols in protocols.io, where a protocol can be assigned its own identifier (DOI) such that it can be cited independently in the future. Additionally, PLOS ONE offers an option to publish peer-reviewed clinical study protocols. Read more information on sharing protocols at <https://plos.org/protocols?utm_medium=editorial-email&utm_source=authorletters&utm_campaign=protocols>
