# Supplementary material for: Epigenetic maintenance of PRC2-repressed chromatin requires RTT109 but not H3K56 acetylation
Source: PLoS Genet. 2026 Oct 5;22(10):e1012327. doi: 10.1371/journal.pgen.1012327 (PMC13652317; doi:10.1371/journal.pgen.1012327)
Supplement: Peer review history

# Peer review: Decision Letter 0

**DOI:** 10.1371/journal.pgen.1012327.r001

**Authors:** Geraldine Butler, Joseph Heitman

29 Jun 2026

PGENETICS-D-26-00550

Epigenetic maintenance of PRC2-repressed chromatin requires RTT109 but not H3K56 acetylation

PLOS Genetics

Dear Zack,

Thank you for submitting this very interesting and well done manuscript to PLOS Genetics.  The manuscript was reviewed by three reviewers, all experts in the field.  You will be glad to hear that they found the work interesting and well done, and suggest minor revisions and some edits to consider alternatives.  Therefore, we invite you to submit a revised version of the manuscript that addresses the points raised during the review process.  The revisions needed constitute minor revision.

Please submit your revised manuscript within two months (it may not take this long). But if you will need more time than this to complete your revisions, please reply to this message or contact the journal office at plosgenetics@plos.org. When you're ready to submit your revision, log on to https://www.editorialmanager.com/pgenetics/ and select the 'Submissions Needing Revision' folder to locate your manuscript file.

Please include the following items when submitting your revised manuscript:

* A letter that responds to each point raised by the editor and reviewer(s). You should upload this letter as a separate file labeled 'Response to Reviewers'. This file does not need to include responses to formatting updates and technical items listed in the 'Journal Requirements' section below.

* A marked-up copy of your manuscript that highlights changes made to the original version. You should upload this as a separate file labeled 'Revised Manuscript with Track Changes'.

* An unmarked version of your revised paper without tracked changes. You should upload this as a separate file labeled 'Manuscript'.

If you would like to make changes to your financial disclosure, competing interests statement, or data availability statement, please make these updates within the submission form at the time of resubmission. Guidelines for resubmitting your figure files are available below the reviewer comments at the end of this letter.

As the corresponding author, your ORCID iD is verified in the submission system and will appear in the published article. PLOS supports the use of ORCID, and we encourage all coauthors to register for an ORCID iD and use it as well. Please encourage your coauthors to verify their ORCID iD within the submission system before final acceptance, as unverified ORCID iDs will not appear in the published article. Only the individual author can complete the verification step; PLOS staff cannot verify ORCID iDs on behalf of authors.

We look forward to receiving your revised manuscript.

All best

Joe

Joseph Heitman, MD, PhD

Academic Editor

PLOS Genetics

Geraldine Butler

Section Editor

PLOS Genetics

Aimée Dudley

Editor-in-Chief

PLOS Genetics

Anne Goriely

Editor-in-Chief

PLOS Genetics

**Additional Editor Comments (if provided):**

**Journal Requirements:**

If the reviewer comments include a recommendation to cite specific previously published works, please review and evaluate these publications to determine whether they are relevant and should be cited. There is no requirement to cite these works unless the editor has indicated otherwise.

1) Please ensure that the CRediT author contributions listed for every co-author are completed accurately and in full.

At this stage, the following Authors/Authors require contributions: Rochelle E. Yap, Felicia Ebot-Ojong, Abigail J. Ameri-Solanki, and Zachary A Lewis. Please ensure that the full contributions of each author are acknowledged in the "Add/Edit/Remove Authors" section of our submission form.

The list of CRediT author contributions may be found here: https://journals.plos.org/plosgenetics/s/authorship#loc-author-contributions

2) We ask that a manuscript source file is provided at Revision. Please upload your manuscript file as a .doc, .docx, .rtf or .tex. If you are providing a .tex file, please upload it under the item type u2018LaTeX Source Fileu2019 and leave your .pdf version as the item type u2018Manuscriptu2019.

3) We do not publish any copyright or trademark symbols that usually accompany proprietary names, eg ©,  ®, or TM  (e.g. next to drug or reagent names). Therefore please remove all instances of trademark/copyright symbols throughout the text, including:

- TM on pages: 31, and 33.

4) Please upload all main figures as separate Figure files in .tif or .eps format. For more information about how to convert and format your figure files please see our guidelines:

https://journals.plos.org/plosgenetics/s/figures

5) We have noticed that you have uploaded Supporting Information files, but you have not included a list of legends. Please add a full list of legends for your Supporting Information files after the references list.

6) Please amend your detailed Financial Disclosure statement. This is published with the article. It must therefore be completed in full sentences and contain the exact wording you wish to be published.

i) State what role the funders took in the study. If the funders had no role in your study, please state: "The funders had no role in study design, data collection and analysis, decision to publish, or preparation of the manuscript."

ii) If any authors received a salary from any of your funders, please state which authors and which funders..

If you did not receive any funding for this study, please simply state: u201cThe authors received no specific funding for this work.u201d

7) We have amended your Competing Interest statement to comply with journal style. We kindly ask that you double check the statement and let us know if anything is incorrect.

**Reviewers' comments:**

Reviewer's Responses to Questions

**Comments to the Authors:**

**Please note here if the review is uploaded as an attachment.**

Reviewer #1: This manuscript identifies RTT109 as a regulator of facultative heterochromatin maintenance in Neurospora crassa and demonstrates that this function is independent of NAF-2 and H3K56 acetylation. The authors combine genetics, transcriptomics, chromatin profiling, protein interaction analyses, and an elegant synthetic recruitment system to investigate how H3K27me3 domains are maintained through mitotic divisions. The work addresses an important question in chromatin biology and provides convincing evidence that RTT109 contributes to maintenance, rather than establishment, of facultative heterochromatin.

The experiments are well designed and the conclusions are generally supported by the data. In particular, the synthetic tetO-SUZ12 recruitment system provides strong evidence that H3K27me3 can be maintained in a PRC1-independent fungal system and that RTT109 is required for this process.

Major comments

1. The authors show that RTT109 regulates facultative heterochromatin independently of NAF-2 and H3K56 acetylation. This result raises an obvious mechanistic question: which molecular form of RTT109 carries out this function? The data exclude the canonical RTT109–NAF-2–H3K56ac pathway, but they do not address whether RTT109 acts as a free protein, in association with NAF-2, or as part of another protein complex. Because RTT109 co-purifies with NAF-2 whereas NAF-2 itself is dispensable for maintenance of facultative heterochromatin, the possibility that RTT109 participates in additional protein complexes deserves consideration. The authors could address this point experimentally using the existing RTT109-3xFLAG strain. Native size-exclusion chromatography or glycerol gradient fractionation followed by anti-FLAG immunoblotting would determine whether RTT109 exists in multiple biochemical pools. Analysis of NAF-2 co-fractionation would help distinguish the canonical RTT109–NAF-2 complex from potential NAF-2-independent assemblies. In addition, RTT109 immunoprecipitation in a naf-2Δ background could establish whether (i) Rtt109 interacts to heterochromatic domains and (ii) this interaction is dependent on its canonical protein complexes.

I do not consider identification of the relevant RTT109 substrate necessary for publication. However, a simple biochemical characterization of RTT109 complexes would strengthen the mechanistic interpretation of the study.

2. The effects of RTT109 loss on facultative heterochromatin-associated H3K36me3 are interesting and may provide insight into the underlying mechanism. The passaging experiments suggest that loss of H3K36me3 occurs before the progressive loss of H3K27me3. The authors should discuss this possibility in greater detail. If feasible, analysis of ASH1 occupancy (by ChIP-seq/ChIP PCR) or ASH1 protein levels in the Δrtt109 strain would help clarify this point.

3. In the introduction, the authors state that RTT109 plays a role in quelling and small-RNA production in N. crassa. However, the discussion does not revisit this aspect of RTT109 biology. The authors should discuss whether the previously reported roles of RTT109 in d small-RNA pathways could be related to the chromatin phenotypes described here, or whether they consider these functions to be independent.

Reviewer #2: The authors present a comprehensive analysis of RTT109 in N. crassa and show that it is required for the establishment of facultative heterochromatin (fHC) at several regions in the genome. The manuscript reports several important novel findings related to the role of RTT109 in the establishment and maintenance of fHC over mitotic divisions. Latter has been tested by the implementation of an innovative system using a synthetic TetR-suz12 fusion which allows to target the PRC2 complex conditionally to a TetO array inserted at the his3 locus. They also demonstrated that the acetyltransferase activity of RTT109 is necessary for its PRC2 recruitment function but, surprisingly, the target is not histone H3K56. Apparently, something else must be acetylated by RTT109 to mediate recruitment of PRC2 components. The identity of this RTT109 target remains elusive. In any case, all necessary experimental controls were considered and the manuscript is well structured. In my view, great work from one of the leading labs in fungal chromatin biology and epigenetics.

There are just a few points for the authors to consider:

1. Fig 1 shows that RTT109 is required for the establishment of H3K27me3 domains and concomitant transcriptional repression but quantification by MACS3 only reveals a mild effect (281 region in WT versus 206 regions in RTT109 KO corresponding to a decrease of 29%). As the affected regions are known, it might be interesting to search for position effect biases that define RTT109-targeted regions (e.g. subtelomeric or pericentric regions bias?). This could be checked and if any significant enrichments appear, amended the manuscript with these data.

2. Related to this possible positional bias are the results presented in Fig 5 in which the role of RTT109 in H3K27me3 maintenance during mitotic divisions was tested. Actually, H3K27me3 deposition at the TetO array inserted at the his 3 locus is not RTT109 dependent. Only the maintenance and mitotic stability is. One interpretation consistent with the data is that RTT109 is necessary only for PRC2 recruitment, but not for its function. If recruitment occurs via TetOFF-suz12, then RTT109 is not necessary anymore (K27me3 equally strong in WT and mutant background, Fig 5 E/F). I think this should be more clearly stated in the discussion. Also, possible candidates that are known to affect H3K27 methylation, could be checked for the acetylation status in the published dataset of Wang et al (2021). PRC2 components were not found, but were other factors known to affect K27 methylation checked?

3. Fig 3 G: the loading of the lanes for the Western blot does not look balanced as the H3 band is much weaker in the rtt109 mutant. I suggest a densitometric analysis of serial dilutions that should allow quantification of H3K27 methylation in the different genetic backgrounds.

Minor points:

The authors should mention in the introduction that H3K27 methylation also regulates secondary metabolite production in fungi and that many filamentous ascomycetes do not contain the PRC2 machinery to establish fHC this way.

Reviewer #3: The study identifies a novel role for the histone acetyltransferase RTT109 in regulating PRC2-mediated facultative heterochromatin. RTT109 is primarily known for its roles in replication-coupled nucleosome assembly and DNA repair through H3K56 acetylation. Its involvement in maintaining H3K27me3-marked facultative heterochromatin was previously unknown, thus expanding its functional repertoire beyond its established roles in euchromatin and DNA damage responses.

Importantly, RTT109 is the main enzyme responsible for H3K56 acetylation in fungi, and most of its known biological functions have been attributed to this modification. The finding that RTT109 maintains H3K27me3 domains through its catalytic activity, yet independently of H3K56 acetylation, therefore suggests a distinct non-canonical mechanism of action. This uncouples its role in facultative heterochromatin from its established H3K56 acetylation-dependent functions.

Furthrermore, although epigenetic inheritance of H3K27me3 across cell divisions is well documented in metazoans, fungi lack the PRC1 complex, which is often critical for this process in higher eukaryotes. The observation that Neurospora crassa can stably propagate H3K27me3 over multiple mitotic divisions therefore provides evidence for a PRC1-independent mechanism for epigenetic memory.

Few additional considerations:

- Maybe this reviewer missed that, but It has not been shown that the tetR-GFP-SUZ12 is fully displaced from the tetO array after tetracycline addition under the conditions used. While tetracycline reliably induces native TetR dissociation through conformational changes, fusion proteins can exhibit altered functional properties compared to their native components, so anti-GFP ChIP-seq followup after tetracycline treatment would be handy.

- This study reports that RTT109 deficiency does not affect constitutive heterochromatin in N. crassa. This result has already been reported in ref. 56.

- It is still formally possible that RTT109-mediated H3K56Ac may be required for H3K27me3 if one considers a model in which two states exist, “H3K56Ac” and “H3K56”, and “H3K56Ac” represents a “not H3K56” state; in this case replacing H3K56 with anything else will effectively generate a state analogous to “H3K56Ac”.

**********

**Have all data underlying the figures and results presented in the manuscript been provided?**

Large-scale datasets should be made available via a public repository as described in the *PLOS Genetics*  data availability policy, and numerical data that underlies graphs or summary statistics should be provided in spreadsheet form as supporting information.

Reviewer #1: Yes

Reviewer #2: Yes

Reviewer #3: Yes

**********

PLOS authors have the option to publish the peer review history of their article (what does this mean?). If published, this will include your full peer review and any attached files.

If you choose “no”, your identity will remain anonymous but your review may still be made public.

**Do you want your identity to be public for this peer review?**  For information about this choice, including consent withdrawal, please see our Privacy Policy.

Reviewer #1: No

Reviewer #2: No

Reviewer #3: No

[NOTE: If reviewer comments were submitted as an attachment file, they will be attached to this email and accessible via the submission site. Please log into your account, locate the manuscript record, and check for the action link "View Attachments". If this link does not appear, there are no attachment files.]

**Figure resubmission:**

**Reproducibility:**

To enhance the reproducibility of your results, we recommend that authors deposit laboratory protocols in protocols.io, where a protocol can be assigned its own identifier (DOI) such that it can be cited independently in the future. Additionally, PLOS ONE offers an option to publish peer-reviewed clinical study protocols. Read more information on sharing protocols at https://plos.org/protocols?utm_medium=editorial-email&utm_source=authorletters&utm_campaign=protocols

# Author comment: Author response to Decision Letter 1

**DOI:** 10.1371/journal.pgen.1012327.r002

17 Sep 2026

Attachment Submitted filename: ResponseToReview_FINAL.docx

# Editor report: Decision Letter 1

**DOI:** 10.1371/journal.pgen.1012327.r003

**Authors:** Geraldine Butler, Joseph Heitman

21 Sep 2026

Dear Zack,

We are pleased to inform you that your revised manuscript entitled "Epigenetic maintenance of PRC2-repressed chromatin requires RTT109 but not H3K56 acetylation" has been editorially accepted for publication in PLOS Genetics. Congratulations!

Before your submission can be formally accepted and sent to production you will need to complete our formatting changes, which you will receive in a follow up email. Please be aware that it may take several days for you to receive this email; during this time no action is required by you. Please note: the accept date on your published article will reflect the date of this provisional acceptance, but your manuscript will not be scheduled for publication until the required changes have been made.

Once your paper is formally accepted, an uncorrected proof of your manuscript will be published online ahead of the final version, unless you’ve already opted out via the online submission form. If, for any reason, you do not want an earlier version of your manuscript published online or are unsure if you have already indicated as such, please let the journal staff know immediately at plosgenetics@plos.org.

In the meantime, please log into Editorial Manager at https://www.editorialmanager.com/pgenetics/, click the "Update My Information" link at the top of the page, and update your user information to ensure an efficient production and billing process. Note that PLOS requires an ORCID iD for all corresponding authors. Therefore, please ensure that you have an ORCID iD and that it is validated in Editorial Manager. To do this, go to ‘Update my Information’ (in the upper left-hand corner of the main menu), and click on the Fetch/Validate link next to the ORCID field.  This will take you to the ORCID site and allow you to create a new iD or authenticate a pre-existing iD in Editorial Manager.

If you have a press-related query, or would like to know about making your underlying data available (as you will be aware, this is required for publication), please see the end of this email. If your institution or institutions have a press office, please notify them about your upcoming article at this point, to enable them to help maximise its impact. Inform journal staff as soon as possible if you are preparing a press release for your article and need a publication date.

Thank you again for supporting open-access publishing; we are looking forward to publishing your work in PLOS Genetics!

Yours sincerely,

Joe

Joseph Heitman, MD, PhD

Academic Editor

PLOS Genetics

Geraldine Butler

Section Editor

PLOS Genetics

Aimée Dudley

Editor-in-Chief

PLOS Genetics

Anne Goriely

Editor-in-Chief

PLOS Genetics

www.plosgenetics.org

BlueSky: @plos.bsky.social

----------------------------------------------------

Comments from the reviewers (if applicable):

The editor appreciates the careful attention to addressing reviews in the revised manuscript. The revised manuscript is accepted. We appreciate your entrusting this fine study to PLOS Genetics.

----------------------------------------------------

**Data Deposition**

If you have submitted a Research Article or Front Matter that has associated data that are not suitable for deposition in a subject-specific public repository (such as GenBank or ArrayExpress), one way to make that data available is to deposit it in the Dryad Digital Repository. As you may recall, we ask all authors to agree to make data available; this is one way to achieve that. A full list of recommended repositories can be found on our website.

The following link will take you to the Dryad record for your article, so you won't have to re‐enter its bibliographic information, and can upload your files directly:

http://datadryad.org/submit?journalID=pgenetics&manu=PGENETICS-D-26-00550R1

More information about depositing data in Dryad is available at http://www.datadryad.org/depositing. If you experience any difficulties in submitting your data, please contact help@datadryad.org for support.

Additionally, please be aware that our data availability policy requires that all numerical data underlying display items are included with the submission, and you will need to provide this before we can formally accept your manuscript, if not already present.

----------------------------------------------------

**Press Queries**

If you or your institution will be preparing press materials for this manuscript, or if you need to know your paper's publication date for media purposes, please inform the journal staff as soon as possible so that your submission can be scheduled accordingly. Your manuscript will remain under a strict press embargo until the publication date and time. This means an early version of your manuscript will not be published ahead of your final version. PLOS Genetics may also choose to issue a press release for your article. If there's anything the journal should know or you'd like more information, please get in touch via plosgenetics@plos.org.

# Editor report: Acceptance letter

**DOI:** 10.1371/journal.pgen.1012327.r004

**Authors:** Geraldine Butler, Joseph Heitman

PGENETICS-D-26-00550R1

Epigenetic maintenance of PRC2-repressed chromatin requires RTT109 but not H3K56 acetylation

Dear Dr Lewis,

We are pleased to inform you that your manuscript entitled "Epigenetic maintenance of PRC2-repressed chromatin requires RTT109 but not H3K56 acetylation" has been formally accepted for publication in PLOS Genetics! Your manuscript is now with our production department and you will be notified of the publication date in due course.

The corresponding author will soon be receiving a typeset proof for review, to ensure errors have not been introduced during production. Please review the PDF proof of your manuscript carefully, as this is the last chance to correct any errors. Please note that major changes, or those which affect the scientific understanding of the work, will likely cause delays to the publication date of your manuscript.

Soon after your final files are uploaded, unless you have opted out or your manuscript is a front-matter piece, the early version of your manuscript will be published online. The date of the early version will be your article's publication date. The final article will be published to the same URL, and all versions of the paper will be accessible to readers.

For Research Articles, you will receive an invoice from PLOS for your publication fee after your manuscript has reached the completed accept phase. If you receive an email requesting payment before acceptance or for any other service, this may be a phishing scheme. Learn how to identify phishing emails and protect your accounts at https://explore.plos.org/phishing.

Thank you again for supporting PLOS Genetics and open-access publishing. We are looking forward to publishing your work!

With kind regards,

Sharmila Kamatchi

PLOS Genetics

On behalf of:

The PLOS Genetics Team

Carlyle House, Carlyle Road, Cambridge CB4 3DN | United Kingdom

plosgenetics@plos.org | +44 (0) 1223-442823

plosgenetics.org | Twitter: @PLOSGenetics
